# Complex left main bifurcation PCI in a patient with dual positivity: A unique interventional approach – A case report

**DOI:** 10.1016/j.ijscr.2025.111115

**Published:** 2025-03-03

**Authors:** H.S. Natraj Setty, Rahul Patil, Chethan Kumar, L. Sridhar, Jayashree Kharge, B.H. Natesh

**Affiliations:** Cardiology Department, Sri Jayadeva Institute of Cardiovascular Sciences and Research, Bangalore, Karnataka, India

**Keywords:** Human immunodeficiency virus (HIV), COVID-19, Percutaneous coronary intervention (PCI), Case report

## Abstract

**Introduction:**

Human immunodeficiency virus (HIV) affects over 38 million people worldwide, with 26 million receiving combined antiretroviral therapy (CART). People living with HIV (PLWH) have a 1.5- to 2-fold increased risk of cardiovascular disease, yet data on coronary revascularization remain limited. The COVID-19 pandemic, caused by severe acute respiratory syndrome coronavirus 2 (SARS-CoV-2), further complicates the management of acute coronary syndrome (ACS) in PLWH. We present a case of an HIV-positive, COVID-19-positive patient with recurrent ACS, requiring urgent percutaneous coronary intervention (PCI) due to hemodynamic instability (systolic BP < 90 mm Hg) and ongoing chest pain despite optimal medical therapy.

**Case presentation:**

A 41-year-old male, positive for SARS-CoV-2 on RT-PCR, presented with effort angina for two months and recurrent acute chest pain for one day. The electrocardiogram (ECG) showed ST-segment elevation. Despite initial medical management, persistent chest pain and hemodynamic instability (BP 86/60 mm Hg, heart rate 110 bpm) necessitated urgent coronary angiography (CAG), revealing significant stenosis. The SYNTAX Score I for our patient was 12, which is favorable for PCI over CABG. The SYNTAX Score II predicted a 4-year mortality risk of 15.3 % for PCI vs. 5.8 % for CABG. A multidisciplinary heart team discussion favored PCI over CABG. The patient underwent double kissing crush (DK crush) bifurcation stenting with drug-eluting stents (DES), achieving TIMI III flow and resolution of symptoms.

**Clinical discussion:**

Coronary revascularization in PLWH is challenging due to a 2- to 3-fold increased risk of myocardial infarction, chronic inflammation, and drug interactions. Additionally, COVID-19 further elevates thrombotic risk. The SYNTAX Score II favored PCI, and considering the patient's unstable condition, PCI was the preferred strategy over CABG to reduce perioperative risks and hospital stay. The DK crush technique is well-documented for complex bifurcation lesions, with studies showing significantly lower target lesion failure rates (4.8 %) than other techniques (10.5 %).

**Conclusion:**

Managing ACS in PLWH with concurrent COVID-19 requires a multidisciplinary approach. Given the limited surgical data, PCI remains a feasible and effective strategy, particularly in hemodynamically unstable patients. Until randomized trials provide definitive guidance, PCI remains the preferred approach for urgent coronary revascularization in PLWH.

## Highlights

• PLWH face challenges in revascularization due to altered pathophysiology and CART effects, with no specific guidelines available.
• COVID-19 complicates ACS management in PLWH, requiring a balance between cardiovascular care and infection control.
• PCI was preferred over CABG based on SYNTAX score, HIV status, and COVID-19, showing its value in high-risk patients.

## 1 Introduction

Human immunodeficiency virus (HIV) has affected >38 million people worldwide, with almost 26 million receiving combined antiretroviral therapy (CART) [1]. There is an overall risk of 1.5–2.0-fold for developing cardiovascular disease in HIV-affected individuals, along with a 4.5-fold increased risk for sudden cardiac death [[2], [3], [4]]. With the widespread availability of CART, the number of people living with HIV (PLWH) is increasing, resulting in more frequent detection of coronary artery disease (CAD) by healthcare professionals in this subset with an increasing number of population needing coronary revascularization, either in the form of percutaneous coronary intervention (PCI) or coronary artery bypass grafting (CABG). The data on revascularisation of HIV patients are scarce, with no specific recommendations for PLWH outside of current guidelines for coronary revascularization [5].

The Coronavirus disease of 2019 (COVID-19), caused by the severe acute respiratory syndrome coronavirus 2 (SARS CoV-2), was first detected in December 2019 and has evolved into a pandemic affecting millions of people worldwide, imposing an unprecedented burden on healthcare systems globally. COVID-19 has resulted in significant changes in healthcare practices, including those in cardiology, necessitating adequate precautions and novel practices from diagnosis to treatment and discharge.

The COVID-19 pandemic has posed numerous challenges ranging from hospitalization in a dedicated ward or isolation in the coronary care unit to the need for personal protection for both patient and the practitioner, making it compulsory for healthcare workers and patients to always wear at least droplets personal protection equipment (PPE) along with surgical mask, gloves, cup, goggles, shoe covers and single-use gown for clinicians, surgical mask and gloves for patient [6].

The COVID 19 has resulted in a reduction in interventional procedures in cardiac catheterization lab activity for both elective and emergency procedures. A survey by the European Association of Percutaneous Cardiovascular Interventions (EAPCI) revealed a reduction in or discontinuation of primary PCI for STEMI by approximately one quarter, along with an increase in the delays to coronary angiography/PCI with a preference for fibrinolysis instead of primary PCI in STEMI [6].

In this case report, we have discussed PLWH with concomitant COVID-19 presenting with recurrent ACS in the form of non-ST elevation myocardial infarction (NSTEMI), presenting with persistent chest pain and hemodynamic instability, unresponsive to medical management, necessitating urgent coronary revascularization with PCI as the preferred mode of revascularisation along with the challenges associated with the procedure.

## 2 Methods

This case report has been prepared following the Surgical Case Report (SCARE) 2023 guidelines to ensure transparency and comprehensive reporting [7].

## 3 Case presentation

**Fig. 1:**
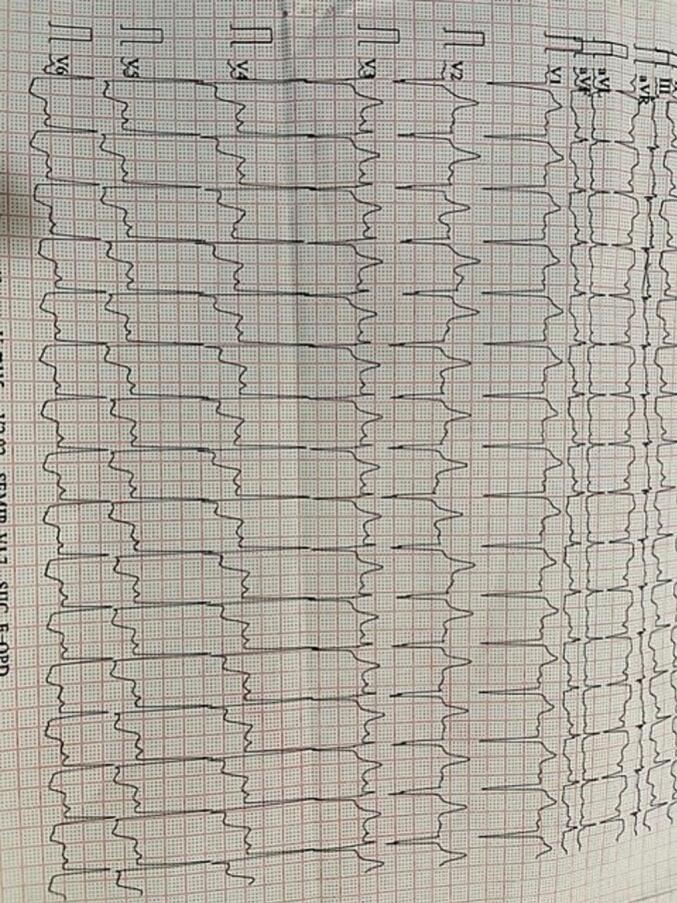
Electrocardiogram (ECG) showing ST segment elevation in lead aVR with ST-segment depressions in the chest leads with.

**Fig. 2:**
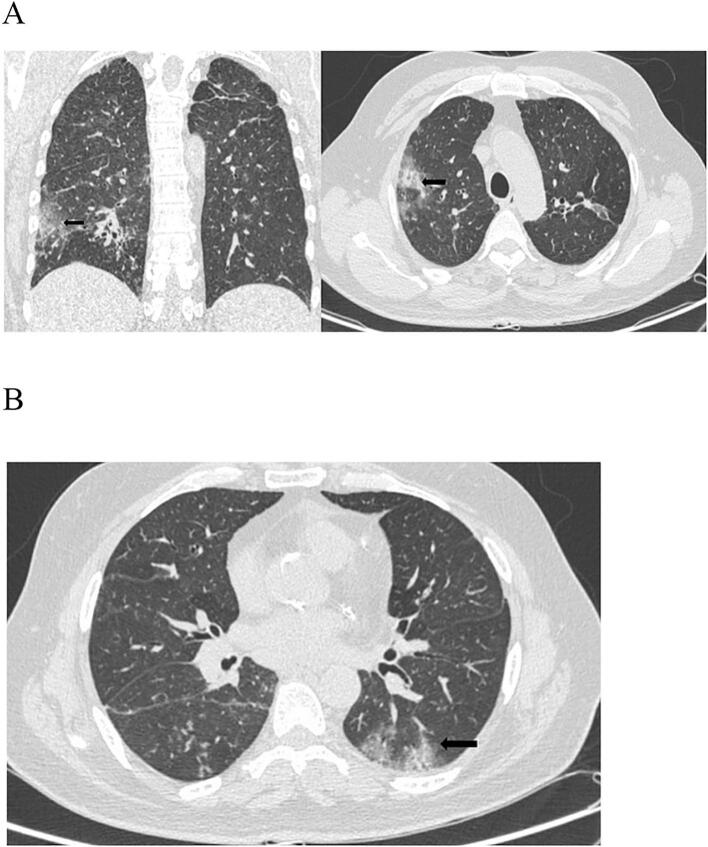
A: Axial non-enhanced CT images (lung window) showing peripheral ground glass opacities in the right upper lobe (arrow). B: Axial non-enhanced CT images (lung window) showing peripheral ground glass opacities in the left lower lobe (arrow).

**Fig. 3:**
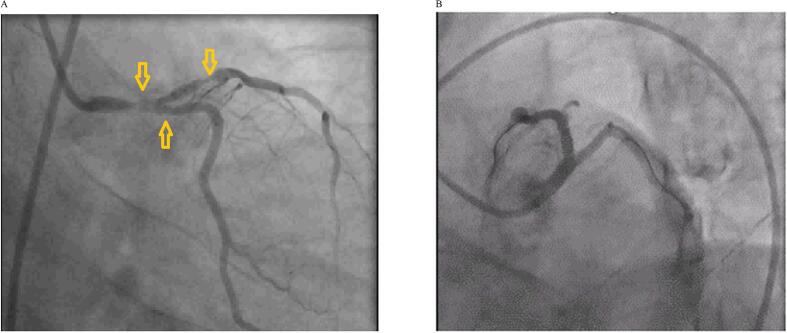
A: Angiography showing Distal LMCA critical stenosis and ostial LAD and LCX stenosis. B: Angiography showing TIMI III Flow -Final Result.

We present a case of a 41-year-old male with positive RT-PCR tests for SARS-CoV-2 and a history of effort angina for the past two months, with recurrent episodes of acute onset chest pain over the past two days. The patient was a known case of HIV on combined antiretroviral therapy (CART), with a CD4 count of 430 cells/μL and an undetectable viral load, indicating good immune control. The patient was classified as WHO Clinical Stage 2. Blood investigations revealed anemia with hemoglobin (Hb) of 8 g/dL and a platelet count of 80,000/μL. After a blood transfusion, Hb improved to 11 g/dL, and the platelet count increased to 1.2 lakh/μL. Liver function tests (LFTs) and renal function tests were normal. However, C-reactive protein (CRP), D-dimer, and ferritin were elevated, indicating an inflammatory state related to COVID-19. An electrocardiogram (ECG) showed ST-segment elevation in lead aVR with ST-segment depressions in chest leads and elevated cardiac troponin T levels [Fig. 1]. Two-dimensional transthoracic echocardiography revealed normal left ventricular size (LV) and contractility with normal valves. Axial non-enhanced CT images (lung window) demonstrated peripheral ground glass opacities in the right upper lobe (arrow) [Fig. 2A] and left upper lobe (arrow) [Fig. 2B]. Initial management included antiplatelets, statins, and unfractionated heparin, but the patient was taken for urgent coronary revascularization due to ongoing chest pain and hemodynamic instability. Coronary angiogram (CAG) [Fig. 3A] revealed 80 % stenosis in the distal left main coronary artery (LMCA), with the left anterior descending artery (LAD) having ostio-proximal disease and distal discrete 50 % stenosis, and the left circumflex artery (LCX) being non-dominant with ostial 70 % stenosis. The right coronary artery (RCA) had a proximal 50 % discrete stenosis, followed by mid 70 % stenosis and distal tubular 80–90 % stenosis, with 80 % stenosis in the ostio-proximal posterior descending artery (PDA) and normal posterior left ventricular artery (PLV).

The SYNTAX Score I was 31, and the SYNTAX Score II predicted a 4-year mortality risk of 15.3 % for PCI and 5.8 % for CABG [8,9]. The EuroSCORE II for our patient was 0.77 %, and the STS Predicted Risk of Mortality (PROM) was 2.33 %, indicating a low surgical risk. However, considering the patient's hemodynamic instability, immunosuppression due to HIV, COVID-19-induced hypercoagulability, and the need for urgent revascularization, CABG posed a higher risk of perioperative complications. Additionally, the SYNTAX Score I was 12 in our patient, which is favorable for PCI over CABG. After a discussion with the heart team, PCI was chosen as the preferred revascularization strategy. The double kissing crush (DK crush) technique was utilized due to its superiority in managing distal LMCA bifurcation lesions [10]. The procedure was completed with a drug-eluting stent (DES) in the mid-LAD (3.0 × 28 mm Xience) followed by two stents at the distal LMCA bifurcation: ostial LCX stenting (3.0 × 23 mm Xience DES) and distal LMCA to proximal LAD stent (4.0 × 23 mm Xience DES), overlapping the previously deployed mid LAD stent. TIMI III flow was achieved [Fig. 3B], and the patient was managed with IABP and mechanical ventilator support. Post-procedure, the patient was started on Remdesivir, Dexamethasone, Tocilizumab, Aspirin, and Ticagrelor, and after intensive management, was extubated after 24 h. The patient showed improvement, with extubation occurring after four days, and was discharged in stable condition. PCI has several potential advantages over CABG, particularly in people living with HIV (PLWH), including less blood loss, fewer transfusions, less risk of blood contact, fewer wound problems, lower risk of pneumonia, shorter hospital stays, and faster recovery [11], with higher adverse events post-CABG compared to PCI [12].

## 4 Conclusion

Coronary revascularisation in PLWH poses novel challenges compared to the general population regarding the pathophysiology of CAD and lack of data guiding decision-making, primarily when associated with COVID-19. Coronary revascularization in the form of PCI is preferable to CABG despite complex coronary anatomy in PLWH, particularly in cases presenting with persisting chest pain and hemodynamic instability wherein immediate revascularisation is required due to short duration of exposure, less risk of blood contact, and early post-procedure recovery with shorter hospital stay associated with PCI. Furthermore, the data on surgical revascularization in PLWH are scarce, and no studies compare CABG to PCI in PLWH. Therefore, coronary revascularisation through PCI is preferred over CABG in this particular subset of patients until conclusive data through randomized studies become available and aid in proper decision-making.

## Consent statement

The patient gave written informed consent for publication and accompanying images. The editor-in-chief of this journal can review a copy of the written consent form upon request.

## Ethical approval

Approved.

## Guarantor

Dr. Natraj Setty H.S

## Research registration number

NA

## Funding

None.

## Author contribution

1. **Dr. H.S Natraj Setty:** Conceptualization, Investigation, Methodology, Project administration, Resources, Visualization, Writing original draft, review & editing
2. **Dr. Rahul Patil:** Formal analysis
3. **Dr. Chethan Kumar:** Investigations
4. **Dr. L Sridhar:** Final Approval of the Manuscript
5. **Dr. Jayashree Kharge:** Data Collection
6. **Dr. Natesh B.H:** Supervision

## Conflict of interest statement

None declared.
